# How clinicians analyze movement quality in patients with non-specific low back pain: a cross-sectional survey study with Dutch allied health care professionals

**DOI:** 10.1186/s12891-017-1649-3

**Published:** 2017-07-04

**Authors:** Margriet van Dijk, Nienke Smorenburg, Bart Visser, Yvonne F. Heerkens, Maria W.G. Nijhuis-van der Sanden

**Affiliations:** 1 HU University of Applied Sciences, Institute for Human Movement Studies, Utrecht, the Netherlands; 2 Faculty of Health, ACHIEVE Centre of Expertise, Amsterdam University of Applied Sciences, Amsterdam, the Netherlands; 3 Research Group Occupation and Health, Nijmegen and Dutch Institute of Allied Health Care, HAN University of Applied Sciences, Amersfoort, the Netherlands; 4 Research Institute for Health Sciences, Scientific Center for Quality of Healthcare, Radboud University Medical Center, Nijmegen, the Netherlands

**Keywords:** Assessment, Allied health care professionals, Low back pain, Movement quality

## Abstract

**Background:**

Observation of movement quality (MQ) is an indelible element in the process of clinical reasoning for patients with non-specific low back pain (NS-LBP). However, the observation and evaluation of MQ in common daily activities are not standardized within allied health care. This study aims to describe how Dutch allied health care professionals (AHCPs) observe and assess MQ in patients with NS-LBP and whether AHCPs feel the need to have a specific outcome measure for assessing MQ in patients with NS-LBP.

**Methods:**

In this cross-sectional digital survey study, Dutch primary care AHCPs (*n* = 114) answered one open and three closed questions about MQ in NS-LBP management. Qualitative and quantitative analyses were applied.

**Results:**

Qualitative analyses of the answers to the open questions revealed four main themes: 1) movement pattern features, 2) motor control features, 3) environmental influences and 4) non-verbal expressions of pain and exertion. Quantitative analyses clearly indicated that AHCPs observe MQ in the diagnostic (92%), therapeutic (91%) and evaluation phases (86%), that they do not apply any objective measurement of MQ and that 63% of the AHCPs consider it important to have a specific outcome measure to assess MQ. The AHCPs expressed added benefits and critical notes regarding clinical reasoning and quality of care.

**Conclusion:**

AHCPs recognize the importance of observing MQ in the assessment and management of LBP in a standardized way. However, there is no consensus amongst AHCPs how MQ should be standardized. Prior to standardization, it will be important to develop a theoretical framework to determine which observable and measurable dimensions of MQ are most valid and relevant for patients with NS-LBP to include in the assessment.

## Background

As in other Western countries, low back pain (LBP) constitutes a considerable health problem in the Netherlands [1]. Dutch patients with LBP are most commonly treated in primary health care by two groups of allied health care professionals (AHCPs): physical therapists (PTs) and exercise therapists (ETs) [2, 3].

In clinical practice, patient complaints about LBP focus on pain and the limitations that it places on regular activities during daily life, work, sports or leisure time [4–6]. The ways in which AHCPs examine features of patient presentations focus on identifying any impairments of body functions and structures, activities limitations and participation restrictions. Moreover, observations of how patients perform daily activities that are problematic due to LBP provide AHCPs with indications for clinical reasoning and for targeting their interventions [7–10]. Nevertheless, the analysis and evaluation of movement quality (MQ), the way in which daily life activities are performed, has yet to be standardized in LBP management [7–11].

Wallbott defines MQ from a psychopathologic perspective as ‘the way in which human movement is executed with respect to the dimensions of time and space’ [12]. In a phenomenological study, PTs developed the Movement Quality Model (MQM), which states that the quality of how a person moves is represented by an interactive process of biomechanical, physiological, psycho-socio-cultural and existential themes [13].

Several reliable observational tools for measuring aspects of MQ in specific target groups are already available. For example, the Body Awareness Rating Scale (BARS) assesses MQ in patients with chronic musculoskeletal disorders and mental health problems. The BARS measurement is based on the observation of 12 movements and a patient interview on movement awareness [14]. The Standardized Mensendieck-Physiotherapy Test (SMT) evaluates MQ in patients with psychosomatic disorders. It assesses functional movement of posture (standing and sitting), movement of upper and lower extremities, gait, and respiration [15]. The Nijmegen Gait Analysis Scale (NGAS) describes and evaluates gait patterns in patients with orthopedic disorders, focusing on the body parts: trunk, pelvis, hip, knee, and ankle [16]. The Functional Movement Screen (FMS) contributes to predicting the risk of sports and occupational injuries to lower extremities. It classifies movement patterns of three functional movements: deep squat, hurdle step and inline lunge. Further, shoulder mobility, active straight leg raise, trunk stability push up and rotary stability are assessed [17–19].

About 90% of all LBP problems are diagnosed as non-specific LBP (NS-LBP). In NS-LBP, no specific pathology (e.g. disc herniation) is identified [20]. The influence of pain on patients with NS-LBP is often observable in adaptations in posture and movement pattern [9]. Compared to people without back pain, patients with NS-LBP adopt consistent movement patterns during the performance of functional activities [21] and tend to move more slowly [22]. Patients with NS-LBP have more rigid pelvis-thorax coordination during gait [23] and have increased lumbar flexion during cycling [24]. Moreover, NS-LPB patients exhibit a specific lumbopelvic pattern during forward bending [25].

In patients with NS-LBP, MQ is described as a multidimensional phenomenon that can be linked to the following components: Body Functions and Body Structures, Activities & Participation, Environmental factors and Personal factors of the ICF (International Classification of Functioning, Disability and Health) [26, 27]. Coordination and functional movement are seen as the most elementary MQ concepts in patients with NS-LBP [26].

Formulating and examining initial hypotheses about how patients with NS-LBP move during activities can help explain their problems [28, 29]. Careful assessment of how they move is required in order to assess specific features of the NS-LBP presentation and create a treatment plan to target these features [9, 11]. In clinical practice, responsive MQ measurement could support the evaluation of MQ changes over time as a result of recovery or intervention [9, 11]. Moreover, the unambiguous description and reliable assessment of MQ could support the realization of multidisciplinary approaches, which are increasingly recommended for such chronic health problems as NS-LBP [30]. An outcome measure for MQ would make it possible to associate changes in how people move with other health indicators (e.g. activity limitations) [11, 22]. This study therefore aims to: 1) elaborate on MQ assessment in clinical practice for patients with NS-LBP and 2) explore the perceived relevance of standardized MQ assessment by AHCPs. The research questions are as follows:

1. How do Dutch AHCPs observe and assess MQ in patients with NS-LBP?
2. Do AHCPs consider it important to have a specific outcome measure for assessing MQ in patients with NS-LBP?

Complementary to our previous study [26], the results of this study will contribute to understanding the relevance of defining and operationalizing MQ in NS-LBP management.

## Method

### Design

We conducted a cross-sectional digital survey with one open and three closed questions to explore how Dutch AHCPs assess MQ in the therapeutic approach for patients with NS-LBP and to obtain their opinions on the importance of having a specific MQ assessment measure. The survey (developed using Thesistools.com) was used to collect data from a large number of anonymous participants.

### Participants

Given that most Dutch patients with NS-LBP are treated in primary health care by PTs and ETs, [2, 3] we included both of these groups of professionals in the study in order to guarantee a broad spectrum of options [31]. We invited AHCPs working in primary care and supervising Bachelor’s level PT and ET students from HU University of Applied Sciences Utrecht and Bachelor’s level ET students from Amsterdam University of Applied Sciences during their internships to participate in this study.

In the first week of November 2010, the secretaries from those universities emailed invitations to all AHCPs supervising internships (385 PTs and 573 ETs). A reminder was sent after six weeks. The email invitations provided information about the aim and procedures of the study and included a link to the digital survey. Interested AHCPs could complete the survey from November 2010 until January 2011. After completing the informed consent, the participants completed a digital questionnaire. Only AHCPs treating patients with NS-LBP in a primary care setting were included.

### Survey

To explore how AHCPs observe MQ in patients with NS-LBP, two researchers (NS and MD) selected four activities that are commonly observed in clinical practice. The selected activities—sitting down and standing up from a chair, lifting, dressing and walking—are the only activities that are included in all three commonly used disability questionnaires: the Quebec Back Pain Disability Scale [32], the Oswestry Low Back Pain Disability Questionnaire [33] and the Roland-Morris Disability Questionnaire [34].

To examine the clarity of the seven survey questions that had been formulated, several colleagues read the survey critically. This small pilot led the researchers to reword two questions and eliminate one.

Survey participants were asked to answer six questions while keeping patients with NS-LBP in mind. This article focuses on Questions III through VI. The results of the analysis of the answers to the first two survey questions (I. ‘*Can you describe the phenomenon of MQ in a few sentences?’* and II. ‘*Please, select the three keywords that you personally consider the most relevant and characteristic for MQ’*) have been described in an earlier article [26].

Question III asked participants to describe, in an open-text field’: *‘What do you observe when you want to analyze whether the movement pattern is adequate during the following activities: a) sitting down and standing up from a chair; b) lifting; c) dressing; and d) walking?’* The participants were asked to describe their observations for each activity separately.

Question IV explored the clinical relevance of MQ by asking: *‘During which phase or phases (diagnostic, therapeutic, evaluation) of your intervention do you observe the quality of the performance of daily life activities in NS-LBP?’* The answer options were ‘diagnostic phase’, ‘therapeutic phase’ and ‘evaluation of the intervention’. Participants could choose one phase or a combination of two or three phases.

Question V explored whether and how AHCPs objectify the observation of daily life activities in their therapeutic approach: ‘*Do you use a measurement instrument to objectify your observation of the quality of the performance of daily life activities in NS-LBP?’* The answer options were ‘yes’, ‘no’ and ‘sometimes’. Participants who answered ‘yes’ or ‘sometimes’ were also asked which instrument they use in their actual practice.

Finally, Question VI aimed to obtain an impression of future needs in order to objectify the observation of MQ: *‘Would you prefer to use a specific observation instrument to assess the quality of performance of daily life activities in NS-LBP?*’ The answer options were ‘yes’ and ‘no’. Participants were further invited to explain their answers in their own words.

### Data analysis

#### Qualitative analysis

An inductive thematic analysis of the answers to Question III was applied in order to identify relevant themes within the data [35]. First, all data items were closely read and thoroughly coded by two independent coders (NS and MD). Both coders are ETs with prior experience in qualitative content analysis [26]. Second, both coders categorized codes that represent identical issues. Third, the research team systematically reviewed the coherence of the identified and categorized codes. Finally, MD grouped the categories into themes that consistently represent all relevant data, after which the themes were discussed with and determined by the research team [31, 36]. The analysis was supported by a computer-assisted system, MAXQDA (Version 10R240113), which made it possible to report calculated frequencies and percentages of the codes contributing to the themes (Table 1).

**Table 1:** Themes, subthemes and illustrating quotes mentioned as aspects of MQ observation

Themes^b^	Subthemes: Observation MQ^a^	Subthemes: Interpretation MQ^a^	Excerpts of the answers to Question I: ‘*I look at ….’*
1. Movement pattern Observation: 469 codes, 46.3% Interpretation: 85 codes, 8.4%	a. Movement pattern (169 codes)		*‘The whole movement’*
	b. Position and interaction of: - pelvis, vertebrae, trunk (115 codes) - lower extremities (75 codes) - upper extremities (29 codes)		*‘Lumbopelvic rhythm’* *‘Also with the relationship between leg, spine, and shoulder positioning’* *‘The amount of the knee flexion’* *‘Position of the shoulders’; ‘Whether the arms swing loosely’*
	c. Posture/alignment (25 codes)		*‘Posture’; ‘Alignment’; ‘Total body position’*
	d. Muscle strength (28 codes)		*‘Whether the patient’s legs or back are engaged in the movement’*
	e. Mobility of the joints (28 codes)		*‘The ability of the joint to perform the specific movement’*
		f. Compensation (40 codes)	*‘Compensatory movements’*
		g. Protection from pain and strain (23 codes)	*‘Prevention of strain’*
		h. Functional movement (19 codes)	*‘Goal achievement’*
		i. Conscious movement (3 codes)	*‘When the patient is aware of the performance’*
2. Motor control Observation: 173 codes, 17.0% Interpretation: 78 codes, 7.7%	a. Coordination (60 codes)		*‘The cooperation of the muscles’*
	b. Balance (45 codes)		*‘I look at balance’*
	c. Speed (44 codes)		*‘Fast, slow’; ‘Speed of the action’*
	d. Symmetry (17 codes)		*‘Asymmetric posture’*
	e. Respiration (7 codes)		*‘How respiration is performed’*
		f. Fluency (78 codes)	*‘Whether the movement is fluent, harmonious, stiff, limber, supple…?’*
3. Environment Observation: 51 codes, 5.0%	a. Used support (35 codes)		*‘Whether the patient uses the armrest of the chair’*
	b. Use of assistive products or help (8 codes)		*‘The use of devices’*
	c. Ergonomics (8 codes)		*‘The distance from the person to the object that was picked up’*
4. Non-verbal expressions Interpretation: 89 codes, 8.8%		a. Pain (39 codes)	*‘Whether someone moves without pain’*
		b. Body language (20 codes)	*‘Facial expressions’*
		c. Exertion (24 codes)	*‘The apparent difficulty of the movement’*
		d. Auditory signs (6 codes)	*‘Verbal expressions of the patient’; ‘What do I hear (*e.g. *sigh, shuffle)?’*

^a^Of four daily life activities: sitting down and standing up from a chair, lifting, dressing and walking; ^b^Frequency (percentage) of 1014 identified codes

*MQ* movement quality, *NS-LBP* non-specific low back pain

Description of non-observable aspects: 69 codes, 6.8%

Text and examples in italics illustrate the content of the answers given. The quotations used were originally in Dutch. The texts were translated by a native English speaker (EF) and verified for their intended meaning by MD.

#### Quantitative analysis and open text analysis

The answers to the closed-ended Questions IV-VI were analyzed using descriptive statistics (calculated frequencies and percentages). The information that AHCPs added to Question V provided insight into the instruments that AHCPs currently apply to objectify the observation of MQ. The motives mentioned in the open text field of Question VI provided insight into intrinsic and extrinsic considerations regarding the importance of MQ as an outcome measure. Intrinsic motives represent the drive of therapists themselves in line with their own therapeutic beliefs or experiences. These motives reflect human propensity to learn and assimilate. [37] Extrinsic motives are driven by external accountability and typify incentives from the environment to realize required professionalism and outcomes with regard to quality of care. [37] Due to differences in the level of details in the motives described, analysis was limited to an enumeration and clustering of the motives. Illustrative quotations (also translated by EF) are provided in italics.

## Results

### Participants

**Fig. 1:**
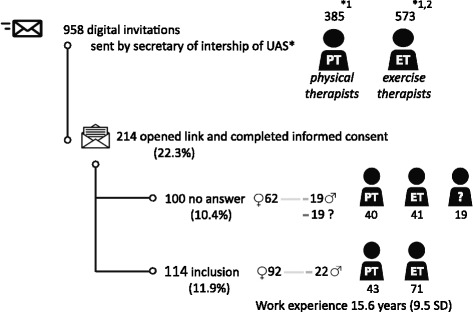
Flow diagram of the participants. * UAS = University of Applied Sciences. 1 = Utrecht, 2 = Amsterdam

As shown in Fig. 1, 214 AHCPs (22.3%) opened the link in the email invitation and completed the informed consent form. One hundred participants (40 PTs, 41 ETs, and 19 with unknown profession) were excluded because they did not answer any of the questions (62 women, 19 men and 19 with unknown gender). All of the remaining 114 AHCPs (11.9%) were included in the analysis. The participants, 43 PTs and 71 ETs, were predominantly women (92, 80.7%). The mean number of years of work experience in treating patients with NS-LBP was 15.6 (9.5 SD).

### Research question 1

#### Observation of MQ (survey question III)

Qualitative analysis revealed four main themes and 22 subthemes, expressing the observations and interpretations of AHCPs with regard to MQ in patients with NS-LBP. The four main themes – 1) movement pattern features 2) motor control features, 3) environmental influences and 4) non-verbal expressions of pain and exertion – and illustrative quotations from the answers are displayed in Table 1. The terms used by the AHCPs refer to the focus of the observation (e.g. ‘*I observe the position of the pelvis during walking’*) and to their interpretation of the performed quality (e.g. *‘Whether someone moves without experiencing pain’*)*.* The subthemes represent observations and the interpretations AHCPs make when analyzing MQ (Table 1). The themes and subthemes cover aspects of MQ that AHCPs described for all four activities: sitting down and standing up from a chair, lifting, dressing and walking (Table 1).

#### Main theme 1: Movement pattern features

More than half of the described *observations* focused on features of movement patterns associated with whole-body activities. When observing movement patterns, AHCPs pay attention to features including: a) the position and interaction of pelvis, vertebrae, trunk and upper and lower extremities (e.g. ‘*Whether the patient’s legs or back are engaged in the movement*’); b) posture/alignment during the activity (e.g. ‘*Posture*’*,* ‘*Alignment*’ or *‘Chain from feet up to the head*’); c) related mobility of the joints (e.g. ‘*The ability of the joint to perform the specific movement*’); and d) muscle strength functions during the activity (e.g. ‘*Whether the used muscle power is sufficient for performing the activity*’)*.* The most detailed observable movement pattern features were described for the activity of walking, including: ‘*Stand and swing phase*’; ‘*Stride length*’; ‘*Foot strike pattern*’; and ‘*Stride width*’*.* The ACHPs often provided detailed descriptions expressing their interpretations of observable qualities of movement pattern features. These interpretations represent four subthemes: e) compensation (e.g. ‘*The degree of compensation related to reduced muscle strength, for example in the upper legs*); f) functional movement (e.g. ‘*Goal achievement*’); g) protection from pain and strain (e.g. ‘*Prevention of strain*’); and h) conscious movement (e.g. ‘*When the patient is aware of the performance*’).

#### Main theme 2: Motor control features

About a quarter of the observations described were related to features that are associated with the control of movements. The motor control features observed by AHCPs when analyzing MQ included: a) coordination (e.g. ‘*The control of the movement*’); b) balance (e.g. ‘*Positioning the center of gravity above the feet*’); c) speed (e.g. ‘*The speed of movement*’); d) movement symmetry (e.g. ‘*Symmetrical bending forward?*’); and e) respiration (e.g. ‘*Control of respiration*’). These features were usually expressed in short sentences (e.g. ‘*I look at balance*’ or ‘*I look at respiration*’).

Descriptions reflecting the interpretations of AHCPs with regard to motor control represent one subtheme – f) fluency – and were expressed with such terms as ‘*Fluency*’*,* ‘*Stiffness*’ *or* ‘*Limberness*’*.*

#### Main theme 3: Environmental influences

A few participants considered environmental influences when observing MQ of whole-body activities. These observations focused on a) the support used (e.g. ‘*Whether the patient uses the armrest of the chair*’); b) the use of assistive products or help from another person and c) ergonomics (e.g. ‘*The position of the feet in relation to the position of the chair*’)*.*

#### Main theme 4: Non-verbal expressions

Quite a few descriptions indicate that AHCPs also use non-verbal expressions and auditory observations to analyze MQ: a) pain (e.g. ‘*Non-verbal signs of pain*’); b) body language (e.g. ‘*Bodily signals*’); c) exertion (e.g. ‘*The effort required to perform the activity?*’); and d) auditory interpretation of the movement performance (e.g. ‘*Plopping down on a chair*’)*.*

A few descriptions expressed non-observable aspects of physical activities (e.g. *‘Insight*’) or outlined the circumstances within which the MQ observation took place (e.g. ‘*Preferably in a large room, possibly with obstacles*’)*.* These data items were excluded from the analysis.

Five participants indicated that the survey question was not clearly formulated.

#### Observation of MQ during treatment and evaluation (survey question IV)

Question IV was answered by 105 AHCPs. The majority of these (*n* = 97, 92.4%) observe MQ during the diagnostic and therapeutic phases, as well as during the evaluation of the treatment. Seven AHCPs (6.7%) observe MQ only during the diagnostic and therapeutic phases, and one therapist (0.9%) observes MQ during the diagnostic phase and the evaluation.

### Research question 2

#### Assessment of MQ (survey questions V and VI)

Quantitative analysis indicated that 71 AHCPs (62%) apply measurement instruments to objectify the observation of MQ of daily life activities, while 38 AHCPs (33%) do not apply measurement instruments to assess MQ. Five AHCPs (4%) did not answer this question. In 114 additional descriptions, 54 AHCPs reported using 21 different measurement instruments for assessing MQ in patients with NS-LBP (Table 2).

**Table 2:** Measurement instruments that AHCPs apply in patients with NS-LBP

Activities & participation^a^ (73, 64%)	Frequency (percentage)
Questionnaires e.g.	45 (40%)
- Roland-Morris Disability Questionnaire	
- Patient Complaints Scale	
Tests e.g.	24 (21%)
- Berg Balance Scale	
- Six Minutes Walking test	
- Time Up & Go test	
Observation list:	4 (3%)
- Nijmegen gait analysis scale	
Body functions^a^ (40, 35%)	
Questionnaires e.g.	34(30%)
- Visual Analogue Scale for Pain	
- Tampa Scale for Kinesiophobia	
- Borg Rating Perceived Exertion	
Tests:	6 (5%)
- Mobility of joint functions	
- Muscle power functions	
Body structures^a^ (1, 1%)	
Pedi-scoliometer	1(1%)

^a^ICF component = International Classification of Functioning, Disability and Health

*AHCPs* allied health care professionals, *NS-LBP* non-specific low back pain

*n* = 54

Of all participating AHCPs, 72 (63%) considered it important to use an instrument to assess MQ, while 34 (30%) did not perceive a need to assess MQ with a specific instrument. Eight AHCPs (7%) did not answer this question. A preference for MQ assessment in the future was expressed by AHCPs who currently did objectify their observation of MQ with a measurement instrument, as well as by those who did not (Table 3).

**Table 3:** Current and future assessment of MQ in patients with NS-LBP

Future	Currently	
	AHCPS *objectify* observed MQ with divergent measurement instruments (71 AHCPs)	AHCPs *do not objectify* observed MQ with any measurement instrument (38 AHCP)
Need	45 AHCPs (39%)	27 AHCPs (24%)
No need	23 AHCPS (20%)	11 AHCPs (10%)
Perceived need is unknown	3 AHCPs (3%)	-

*MQ* movement quality, *NS-LBP* non-specific low back pain, *AHCPs* allied health care professionals

Five AHCPs (4%) did not describe their usage and future preference regarding MQ assessment

In response to Survey Question VI, AHCPs expressed both intrinsic and extrinsic motives. We identified 92 intrinsic motives (80%) and 23 extrinsic motives (20%) that were described by 59 AHCPs with a preference for MQ assessment in the future (proponents) and by 29 AHCPs who did not perceive the need to assess MQ with a measurement instrument (opponents).

#### Intrinsic motives

The intrinsic motives of *proponents* (61 motives, 53% of all motives mentioned) expressed both added benefits and critical notes with regard to MQ assessment. According to these participants, MQ assessment in patients with NS-LBP provides clarity and objectivity for clinical reasoning (26 motives, 23%) (e.g. ‘*Because it sometimes becomes more clear to the patient*’ and ‘*Objectifying the judgment*’). The AHCPs further described that MQ assessment is an adequate addition to existing clinimetrics (8 motives, 7%) (e.g. ‘*Existing instruments are primarily quantitative*’. Additional motives (e.g. ‘*It’s easier to indicate whether and to what extent progress has been made*’) indicate the relevance of MQ as an outcome measure for evaluating treatment (20 motives, 17%).

Critical notes regarding the clinical reasoning of the proponents (7 motives, 6%) expressed that MQ assessment is not necessary for clinical reasoning (e.g. ‘*To me personally, it is not important*’).

The intrinsic motives of the *opponents* (28 motives, 24%) also emphasized that, as an outcome measure, MQ does not contribute to clinical reasoning (e.g. ‘*Observing the manner of movement provides an adequate impression*’). In contrast, three motives (3%) emphasized benefits to clinical reasoning (e.g. ‘*I think it would be good if the manner of observation could be more uniform, in order to achieve a qualification of an activity that is comparable to that of other professionals*’).

#### Extrinsic motives

All extrinsic motives expressed that the use of a specific outcome measure to assess MQ provides insight into the quality of care for others involved. This was expressed by both proponents (21 motives, 18%) (e.g. ‘*It contributes to improving the measurement of the quality of care delivered*’) and opponents (2 motives, 2%) (e.g. ‘*This is necessary for demonstrating the value of our therapeutic approach towards others, including referrers, colleagues and insurance companies*’)*.*

## Discussion

This mixed-method study is intended to identify how Dutch AHCPs observe and assess MQ in the daily activities of patients with NS-LBP. It also explores the opinions of AHCPs regarding the importance of having a specific outcome measure to assess MQ in patients with NS-LBP. The results clearly indicate that MQ plays a central role in NS-LBP management. When analyzing MQ, AHCPs make observations and interpretations of features of movement patterns and motor control, taking into account environmental influences and non-verbal expressions of pain and exertion. Although many therapists emphasize the importance of assessing MQ in patients with NS-LBP, the arguments of AHCPs differ with regard to having a specific outcome measure to assess MQ.

To the best of our knowledge, this is the first study to describe how Dutch AHCPs observe and assess MQ when treating patients with NS-LBP. The results of both qualitative and quantitative analyses clearly expose various perceptions of AHCPs with regard to MQ in such activities as sitting down and standing up from a chair, lifting, dressing and walking. The diversity in the ways in which AHCPs observe, describe and interpret MQ corresponds to the finding that AHCPs implicitly determine and evaluate MQ. Nevertheless, therapists do not routinely choose specific measurement instruments [38]. Further, AHCPs hold differing views and arguments concerning the importance of assessing MQ in the daily activities of patients with NS-LBP. The discrepancy between how AHCPs act (implicit analysis of MQ) and think (a majority of the AHCPs emphasize the importance for using a specific outcome measure to assess MQ) has been recognized in the literature [39, 40]. The divergent approaches and perspectives of AHCPs with regard to MQ might arise from divergent theoretical constructs and therapeutic beliefs [41, 42]. The variability in approach and perspectives in the answers of PTs and ETs was large. Both groups substantially contribute to NS-LBP management in primary care [2, 3] and use comparable LBP guidelines [8, 10]. Therefore, we decided to analyse the groups PTs and ETs, as one group in this study. However, we cannot rule out that individual knowledge, experience and professional background influenced interpretations for clinical reasoning. Therefore, future research needs to discover if professionals differ in the interpretation of the MQ in clinical reasoning for instance by using standardised patients.

This study is subject to several limitations. First, the external validity of this study is limited by a low response rate – a problem that is commonly associated with survey studies [36]. Nevertheless, many PTs and ETs did take the effort to reflect on their therapeutic approaches and to answer the questions.

Second, although we did conduct a small pilot to determine the clarity of the survey questions, some bias occurred. Five AHCPs expressed that they did not clearly understand Question III. With respect to Question V, AHCPs (62%) replied that they use a measurement instrument to objectify their observation of MQ. With the exception of one standardized observation list for gait [16], the AHCPs reported using questionnaires and performance tests to objectify MQ. A closer look at the reported outcome measures give the impression that it is unlikely that information on MQ can be determined based on these instruments. The questionnaires focus on possible difficulties with daily activities and the performance tests might be useful for standardised observation. However, the reported outcome measures of these tests are not necessarily related to MQ. It is possible that AHCPs mentioned measurements used in NS-LBP not specific related to MQ, because they interpreted question V slightly different or they presented what they had. We hypothesize the last, because in their answers to Question III, the AHCPs provided a clear indication of what they observe in order to analyze the adequacy of movement patterns during daily activities. For example, detailed observable movement pattern features for the activity of walking included: ‘*Stand and swing phase*’, ‘*Stride length*’, ‘*Foot strike pattern*’, and ‘*Stride width*’*.* The majority of the AHCPs provided additional information on the closed-ended Question VI, which enhanced the quality of the data and the meaning of the answers [31, 36]. Taken together, we need to conclude that the interpretation of question III and V leads to some bias in the results. However, apparently the observations of MQ are performed alongside tests to measure performance. This can be used in the development of a MQ assessment.

Third, the acuteness of NS-LBP problems is a vital aspect in patient presentations and therapist interpretations. Nevertheless, Question III did not differentiate between observations of activities in patients with acute or chronic NS-LBP. We identified four main themes that AHCPs observe in order to analyze MQ. These themes thus indicate only the points that are of interest to the analysis of MQ in NS-LBP, and difference between acute or chronic stages of NS-LBP were not mentioned. Standardization in the analysis of MQ may have added value in the recognition of different stages of NS-LBP.

Finally, due to the variety of ways in which AHCPs observe and interpret MQ, as well as in their implicit assessments – we decided simply to list the motives, even though the data might be suitable for qualitative content analysis. The dominance of intrinsic motives for having a specific MQ outcome measure nevertheless provides a base for the scientific elaboration of MQ in NS-LBP management and for educational purposes.

Supplementary to our previous study [26], this clinical-practice inventory demonstrates the multidimensionality of MQ in patients with NS-LBP. The observations that AHCPs make of MQ exhibit a number of similarities with three of the four themes of the MQM: biomechanical, physiological and psycho-socio-cultural. The attention that AHCPs pay to features of movement patterns, motor control and the environment is related to the biomechanical theme of the MQM: space, postural stability, path and form. The observations that AHCPs make with regard to motor control features is related to the physiological theme: breathing, flow, elasticity and rhythm [13]. The ‘centering’ character of the physiological theme of the MQM [13] was not evident in the ways in which AHCPs observe MQ. Their observation of non-verbal expressions of pain and exertion corresponds to the psychosocial-cultural theme of the MQM [13]. They did not relate the observation of MQ to the existential theme of the MQM: the person- or self-awareness. It is interesting to note that, although the MQM defines awareness of the body during movements and self-awareness as elementary preconditions of MQ, only a few AHCPs referred to ‘*Conscious movement’* [13]. Awareness and self-awareness did not emerge in their responses. In the development of the MQM, Skjaerven and colleagues (2008) explored essential features and characteristics of MQ in general. In our study, we specifically determined how AHCPs observe and assess MQ in patients with NS-LBP. This might explain why our results contained no evidence of the existential theme and the characteristics of the physiological theme, and why and the participants paid less attention to awareness. ‘Speed’, which was described as a qualitative motor control feature that AHCPs observe to analyze MQ in patients with NS-LBP, seems consistent with the concept of ‘time’ within Wallbott’s concept of MQ [12].

The themes identified in this study reflect how AHCPs observe MQ in the activities of sitting down and standing up from a chair, lifting, dressing and walking. These daily activities often limit functioning in patients with NS-LBP [5, 6, 43, 44]. The observations and interpretations that AHCPs make in order to analyze MQ in daily activities exhibit similarities with the qualitative movement aspects assessed in existing observational tools for MQ. For example, the BARS measures compensations and movement awareness in general movement habits (e.g. symmetrical and asymmetrical stretching in a lying position, flexing/extending the trunk in a standing position, and walking in a circle) [14]. Comparably, AHCP interpretations of qualitative movement pattern features focus on ‘compensation’ and ‘conscious movement’. The observations that AHCPs make with regard to movement patterns features (e.g. ‘position and interaction of pelvis, vertebrae, trunk and upper and lower extremities’) and motor control features (e.g. ‘respiration’) exhibit similarities with the motor functions that are assessed by the SMT [15]. Comparable with the NGAS [16], AHCPs observation of the activity walking focus on the position of the trunk, pelvis, hip, knee, and ankle during gait. The FMS classifies functional movement, fundamental mobility and stability and fundamental core strength and core stability [17, 18]. AHCPs observations regarding movement patterns features (e.g. ‘muscle strength’ and ‘mobility of the joints’) and motor control features (e.g. ‘balance’ and ‘symmetry’) exhibit similarities with the FMS.

The explication of clinical findings promotes the transparency of clinical reasoning, thereby contributing to the development of practical knowledge [41]. Detailed descriptions of the reasoning of PTs could provide more meaningful understanding of physical therapy treatments [41, 45]. Specific MQ assessment is advised, as observation of movement performance is a prerequisite for diagnostic verification and contributes to prioritization and the evaluation of treatment goals and therapeutic approaches [8–11]. Enhanced understanding and clear description of MQ are likely to create opportunities for interdisciplinary approaches, which are increasingly being recommended for the care of people with chronic health problems, including NS-LBP [30]. It could also create the possibility of relating MQ as an outcome to other health indicators [11].

The extensive analysis of MQ during the therapeutic approach supports the idea that MQ plays a central role in clinical reasoning in the therapeutic approach [8–11, 26, 46], and it corresponds to Knudson’s Qualitative Diagnosis of Human Movement (QDM) [42]. The QDM focuses on preparation (knowledge of the activity, goal and performer), observation, diagnosis, evaluation and intervention in sports and exercise [42]. The broad view on analyzing MQ in patients with NS-LBP might be influenced by the principle that the clinical reasoning of AHCPs concentrates on functioning and disability, as well as on contextual factors [4, 8, 10, 46].

To analyze the MQ of activities (e.g. sitting down and standing up from a chair and lifting) in patients with NS-LBP, AHCPs primarily observe features of movement patterns and motor control. At the same time, they interpret qualitative features of movement patterns (e.g. ‘*Compensatory movements*’) and motor control (e.g. ‘*Fluent movement*’) in their clinical reasoning. Experienced PTs base their clinical reasoning largely on pattern recognition [41, 45]. This is the case in both forward and backward reasoning [45]. Interpretations of body movements are also influenced by therapeutic experience [40]. Given that the participants in our study had an average work experience of 15.6 years (SD 9.5), they could be considered experienced. As in our previous study, this result could have been due to the intertwining of observations and interpretations with regard to MQ.

In analyzing MQ, AHCPS pay little attention to environmental factors. This is consistent with findings of our earlier study [26]. As stated by Kirschneck and colleagues (2010), few PT interventions are directed at environmental factors [4].

As in our previous study, AHCPs link pain to MQ in patients with NS-LBP [26]. To analyze MQ, AHCPs observe non-verbal expressions of pain and exertion (e.g. facial expressions and body language). They also note auditory signs (e.g. sighing or plopping down on a chair). These non-verbal expressions correspond to direct observations of pain behavior (e.g. guarding, bracing, rubbing, grimacing and sighing) [47]. Pain behavior is related to position and movement in patients with LBP [47, 48], and it is regarded as an influential factor for chronic LBP and disability [47, 49]. Valid judgments of facial expressions and pain must obviously be included in the development of a standardized method for assessing MQ. It is conceivable that AHCP interpretations of motor control observations (e.g. ‘stiff movement’ or ‘conscious movement’) could also be seen as guarding (e.g. abnormally stiff, interrupted or rigid movement). The distinction of pain behavior should therefore be taken into account during the observation of MQ.

Given the multidimensionality of MQ [12, 13, 26, 42], the variety of ways in which AHCPs observe and interpret MQ, the implicit objectification of AHCPs and the growing integration of functional movement in NS-LBP management [7, 9, 22, 44], we endorse the need to develop a suitable method for measuring and evaluating functional movement in patients with NS-LBP [9, 11, 21].

## Conclusions

This clinical-practice inventory elaborates the central role of MQ in NS-LBP management. The results demonstrate the variety of ways in which AHCPs observe and interpret MQ, as well as the implicit way in which they objectify MQ. AHCPs recognize the importance of assessing MQ in those with NS-LBP although their arguments regarding the benefit for clinical reasoning diverge. Prior to standardization, it will be important to develop a theoretical framework aimed at determining which key functional problems and measurable dimensions of MQ belong in such a standardized MQ assessment method for patients with NS-LBP. The framework should be based on a thorough review of the literature, and the perspectives of therapists, patients and movement scientists must be considered [50].

## Abbreviations

AHCPs: Allied health care professionals
BARS: Body Awareness Rating Scale
ETs: Exercise therapists
FMS: Functional Movement Screen
ICF: International Classification of Functioning, Disability and Health
LBP: Low back pain
MQ: Movement quality
MQM: Movement Quality Model
NGAS: Nijmegen Gait Analysis Scale
NS-LBP: Non-specific low back pain
PCS: Patients Complaints Scale
PTs: Physical therapists
QBPDS: Quebec Back Pain Disability Scale
SMT: Standardized Mensendieck-Physiotherapy Test
UAS: University of Applied Sciences
